# Dual target gene therapy to EML4-ALK NSCLC by a gold nanoshell-based system: Erratum

**DOI:** 10.7150/thno.43075

**Published:** 2020-01-18

**Authors:** Siwen Li, Yuxi Liu, Yalan Rui, Liping Tang, Samuel Achilefu, Yueqing Gu

**Affiliations:** 1 Department of Biomedical Engineering, School of Engineering, China Pharmaceutical University, 24th Tong Jia street, Nanjing 210009, Jiangsu Province, China. Phone: 86-25-83271046; Fax: 86-25-83271046.; 2 Department of Bioengineering, University of Texas at Arlington, Arlington, TX, USA.; 3 Department of Radiology, School of Medicine, Washington University in St. Louis, MO, USA

In our paper 1, Figure 1 and Figure 5 should be corrected as follows.

The corrected figures do not affect the original conclusions of the findings.

## Figures and Tables

**Figure 1:**
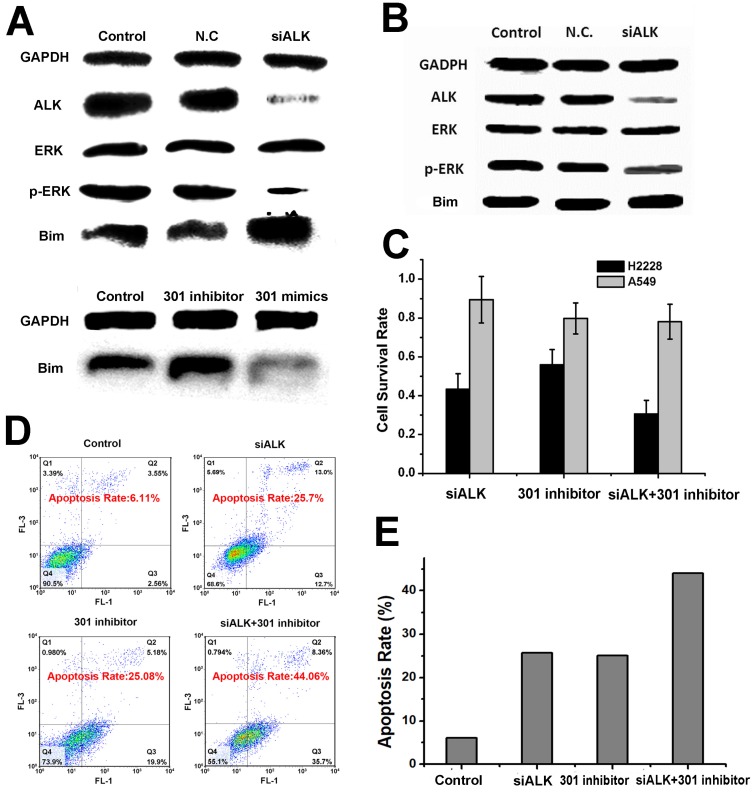
** The mechanism of treatment target selection and the synergistic effect of gene silencing. (A)** Western blot assay after ALK siRNA, miR-301 inhibitor and miR-301 mimics transfection in H2228 cells. **(B)** Western blot assay after ALK siRNA transfection in A549 cells. **(C)** Survival of cells treated under the conditions in (A-B) determined by MTT assay. **(D)** Apoptosis of cells treated under the above conditions assayed by AV-PI kit. **(E)** Quantitative analysis of (D). Data are given as mean ± SD (n=5). *, P<0.05.

**Figure 5:**
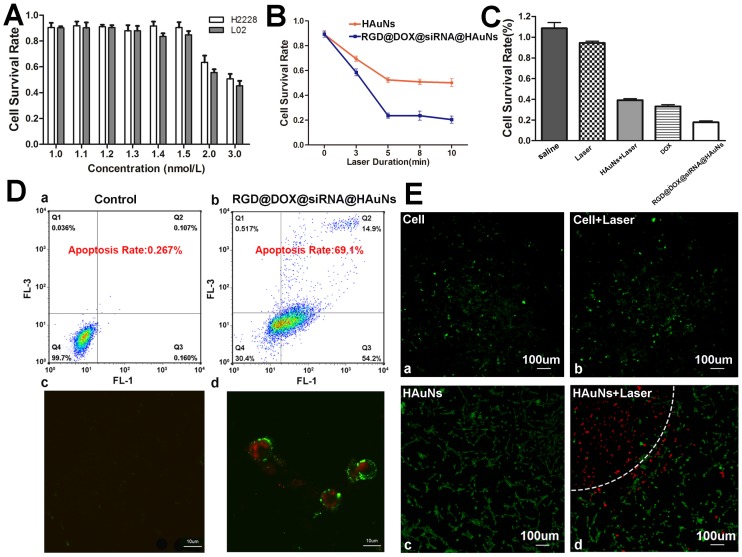
** Treatment efficacy of RGD@siRNA@MPA@HAuNs at a cellular level. (A)** Survival rate of H2228 cells and L02 cells after incubating with different concentrations of bare HAuNs. **(B)** Survival rate of H2228 cells after incubating with bare HAuNs and RGD@siRNA@MPA@HAuNs with different laser duration times. **(C)** Survival rate of H2228 cells treated under the above conditions determined by MTT assay. **(D)** Flow cytometry and laser confocal microscopy examination of the apoptosis of H2228 cells after irradiation of RGD@siRNA@MPA@HAuNs. **(E)** Laser confocal microscopy of photothermal therapy of HAuNs in H2228 cells; viable cells were stained green with Calcein-AV, and dead cells were stained red with PI. Cells were treated with only the dye (a) as the control group, HAuNs without irradiation (b), irradiation only (c) and HAuNs with irradiation (d). Data are given as mean ± SD (n=5). *, P<0.05.
